# The potential role of gut microbiota in the occurrence and development of endometriosis

**DOI:** 10.3389/fcimb.2024.1454931

**Published:** 2024-10-31

**Authors:** Jing Guo, Xianyue Yan, Liping Han

**Affiliations:** ^1^ Department of Gynaecology and Obstetrics, The First Affiliated Hospital of Zhengzhou University, Zhengzhou, Henan, China; ^2^ Department of Gynaecology and Obstetrics, Zhengzhou Central Hospital Affiliated to Zhengzhou University, Zhengzhou, Henan, China; ^3^ Department of Gynaecology and Obstetrics, Henan Provincial People’s Hospital, Zhengzhou, Henan, China

**Keywords:** endometriosis, gut microbiota, immunity, biomarker, proteobacteria

## Abstract

Endometriosis (EMT) has a significant impact on women’s physical and mental health. In this study, high-throughput sequencing technology was employed to detect differences in gut microbiota between EMT patients and healthy individuals (CTL). Additionally, Spearman correlation analysis was utilized to analyze the correlation between different bacterial genera and EMT biomarkers (CA125 and CA199). The results demonstrated that at the phylum level, the relative abundances of Proteobacteria and Desulfobacterota_G_459546 in the EMT group were significantly higher than those in the CTL group, while the relative abundances of Bacteroidota and Firmicutes_A in the EMT group were significantly lower than those in the CTL group. At the genus level, the relative abundances of *Burkholderiales* and *Sphingomonadales* in the EMT group were significantly higher than those in the CTL group, while the relative abundances of *Bacteroidales* and *Roseburia* in the EMT group were significantly lower than those in the CTL group. The correlation analysis results show that CA125 and CA199 are significantly positively correlated with *Burkholderiales* and *Sphingomonadales*, and significantly negatively correlated with *Bacteroidales*, *Oscillospirales*, and *Roseburia*. The PICRUSt2 results show that the relative abundance in the cell motility and xenobiotics biodegradation and metabolism pathways in the EMT group was higher than that in the CTL group, while the relative abundance in the translation, replication and repair, folding, sorting and degradation, metabolism of terpenoids and polyketides and metabolism of cofactors and vitamins pathways in the EMT group was lower than that in the CTL group. In brief, there is a close correlation between the imbalance of gut microbiota and the onset of EMT. The intestinal microbiota has great significance broad prospects for the prevention, diagnosis and treatment of EMT.

## 1 Introduction

Endometriosis (EMT), which refers to the appearance of endometrial tissue (glands and stroma) in locations other than the uterine body and is called EMT, is a common gynecological disease (Vercellini et al., 2014; Wang et al., 2022). The main symptom is pain, and it can cause infertility, seriously affecting the physical and mental health of women (Giudice and Kao, 2004). Epidemiological investigations reveal that the incidence of EMT is approximately 5% to 15%, and 30-50% of women suffer from infertility (Jurkiewicz-Przondziono et al., 2017). In recent years, the incidence rate has shown an upward trend, which is positively correlated with the social and economic situation and is related to the increase of cesarean section, hysteroscopy, and artificial abortion operations (Medina-Perucha et al., 2022). In 1885, Von Rokitansky first described EMT, and over a century has passed since then (Benagiano and Brosens, 2006; Gunther and Walker, 2024). In this nearly hundred-year history, people have gradually realized that EMT is not just a medical problem, affecting the physical and mental health of women of childbearing age; a series of social problems caused by it are also of concern (Kvaskoff et al., 2021; Sims et al., 2021). Moreover, the diagnosis and treatment of EMT have gone through an extraordinary development process, but up to now, many problems related to EMT are still world problems (Chapron et al., 2019; Rolla, 2019). For example, regarding the cause of EMT, from the classic menstrual blood reflux theory proposed by Sampson in 1921 to the “*in situ* endometrium determination theory” proposed by Jinghe Lang, and then to the “source treatment”, the cause of EMT is still unclear and difficult to explain with a single model (Mehedintu et al., 2014; Chen et al., 2019; Ding et al., 2022). EMT, as a disease like a mystery, involves various fields of gynecology such as gynecological inflammation, endocrinology and tumor (Murakami et al., 2020; Taylor et al., 2021; Wang et al., 2022; Marquardt et al., 2023). In recent years, there have been a large number of studies on EMT (Greene et al., 2016). Genes, epigenetics, immunity, angiogenesis, inflammation, exosomes, and immune checkpoint blockade therapy are the current domestic and international research hotspots of EMT, but most of them are based on experimental animals, and lack clinical research evidence (Czyzyk et al., 2017; Makiyan, 2017; Symons et al., 2018; Zondervan et al., 2020; Li et al., 2023).

Research has demonstrated that the number of intestinal microorganisms is ten times that of human cells (Chung et al., 2012). The intestinal microbiota is intimately related to the body’s immunity (Zheng et al., 2020). Intestinal microorganisms possess anti-tumor and anti-aging properties. Moreover, maintaining a balanced intestinal microbiota can enhance the digestive and absorption functions of the gastrointestinal tract (Villéger et al., 2019; Di Tommaso et al., 2021; Ling et al., 2022). Intestinal microorganisms play an important role in the process of inducing and training the maturity of the body’s immune system. A dynamic balance system is established between the body’s immune system and the intestinal micro-ecosystem (Fu et al., 2022, 2023). Alterations in the body’s immune system can modify the composition of the intestinal microbiota. Conversely, changes in the intestinal microbiota can regulate the body’s immune system and the two interact with each other (D'Amelio and Sassi, 2018; Zhou et al., 2020). EMT is a complex immune disease, accompanied by abnormal functions and quantities of immune cells in the local lesion and abdominal cavity, as well as abnormalities in key cytokines. The disorder of the immune system will impact the composition of intestinal microorganisms (Laschke and Menger, 2016). Therefore, if the influence of intestinal micro-ecology on the immune system of EMT patients can be clearly defined, perhaps it is possible to improve or even correct the disorder of the body’s immune system by intervening in the composition of the intestinal microbiota, thereby enhance the body’s ability to fight EMT, and then screen out new treatment methods for EMT.

In recent years, research on EMT and intestinal micro-ecology has been on a gradual upswing. However, the majority of these studies are animal-based, while there are relatively few investigations on the differences between the intestinal micro-ecology of patients with EMT and that of healthy individuals in the humans (Li et al., 2019; Park et al., 2022). Hence, in this project, starting from the perspective of intestinal micro-ecology, non-invasive fecal specimens are utilized. The intestinal micro-ecology of EMT patients is detected through high-throughput sequencing technology to observe the characteristic changes in the intestinal microbiota of EMT patients. Additionally, comparisons are made with the diversity and structural characteristics of intestinal micro-ecology between normal healthy people. This study holds certain guiding significance for clinical practice. This study can also provide clues for further improving the clinical strategy for EMT. For instance, through fecal laboratory tests, it can provide a basis for determining the presence of EMT based on the characteristics of fecal microbiota. Moreover, whether methods such as using probiotics, nutritional therapy, and fecal microbiota transplantation can improve the symptoms of EMT and reduce lesions also provides new ideas for future research on EMT.

## 2 Materials and methods

### 2.1 Research subjects and grouping

The patients with EMT who were treated in the First Affiliated Hospital of Zhengzhou University and Zhengzhou Central Hospital Affiliated to Zhengzhou University from June 2020 to January 2021 were taken as the research objects. There were 2 groups in this study. 22 patients with EMT were in the research group (EMT group), and 18 healthy people undergoing physical examination were used as the control group (CTL group).

### 2.2 Ethical review

The clinical research plan meets the ethical standards and has been approved by the clinical ethics committee of the scientific research project of the First Affiliated Hospital of Zhengzhou University: 2020L08561.

### 2.3 Inclusion and exclusion criteria

#### 2.3.1 Patients with EMT

1) Age: Reproductive age; 2) Gender: Female; 3) Ethnicity: Han nationality; 4) Having secondary dysmenorrhea which is progressively aggravated, infertility or chronic pelvic pain, gynecological examination reveals a cystic mass connected to the uterus or a tender nodule in the pelvic cavity, or diagnosed by color Doppler ultrasound, or those who have been diagnosed by laparoscopy in the past; 5) Exclusion criteria: Recent digestive system symptoms such as constipation, diarrhea, and bloody stools; Presence of hepatobiliary system and gastrointestinal diseases; History of gastrointestinal surgery; Dysfunction of other organs; Obesity, diabetes and other endocrine system diseases; Having taken antibiotics, gastrointestinal motility drugs, acid-suppressing drugs, intestinal microecological live bacteria preparations, etc. within the past 4 weeks; Have a history of mental illness.

#### 2.3.2 Healthy population

1) Age: Reproductive age; 2) Gender: Female; 3) Ethnicity: Han nationality; 4) Exclusion criteria: Patients with EMT; Recent digestive system symptoms such as constipation, diarrhea, and bloody stools; Presence of hepatobiliary system and gastrointestinal diseases; History of gastrointestinal surgery; Dysfunction of other organs; Obesity, diabetes and other endocrine system diseases; Having taken antibiotics, gastrointestinal motility drugs, acid-suppressing drugs, intestinal microecological live bacteria preparations, etc. within the past 4 weeks and Having a history of mental illness.

### 2.4 Serum marker detection

For patients with EMT and CTL group individuals, a disposable blood collection needle and a negative pressure blood collection tube were used to collect 5 mL of blood on an empty stomach in the early morning. The blood samples collected from the EMT patient group were all taken before treatment. After the blood collection was completed, the blood samples were sent to the hospital laboratory for further testing within 1 h. The levels of serum CA125 and CA199 were determined by electrochemiluminescence immunoassay. The Elesys1010 fully automated electrochemiluminescence analysis system and supporting reagents (Roche, Germany) were used.

### 2.5 Fecal sample collection and DNA extraction and detection

Collect the first morning feces from patients with EMT and people in the healthy physical examination population. Use the Tiangen Stool DNA Kit to extract the fecal microbial genomic DNA following the kit instructions. Use 0.8% agarose gel electrophoresis to detect the size of DNA molecules and an ultraviolet spectrophotometer to quantify the DNA.

### 2.6 Amplification of fragment of bacterial 16S rRNA gene, library construction and sequencing on the machine

Using the extracted genomic DNA as a template, PCR amplification was carried out with specific primers with Barcode and Takara Ex Taq high-fidelity enzyme, targeting the V3-V4 variable region of the bacterial 16S rRNA gene. The universal primers 343F (5’-TACGGRAGGCAGCAG-3’) and 798R (5’-AGGGTATCTAATCCT-3’) were used to amplify the V3-V4 variable region of the 16S rRNA gene, which was used for bacterial diversity analysis. The PCR product was quantified on a Microplate reader (BioTek, FLx800) using the Quant-iT PicoGreen dsDNA Assay Kit, and then the TruSeq Nano DNA LT Library Prep Kit of Illumina was used for library construction, and the library was quality inspected on the Agilent 2100 Bioanalyzer machine using the Agilent High Sensitivity DNA Kit. For the qualified libraries, 2 × 250bp paired-end sequencing was carried out on the NovaSeq machine using the NovaSeq 6000 SP Reagent Kit (500 cycles).

### 2.7 Preprocessing of off-machine data

After sequencing, the following steps are taken to process the raw data. The raw data obtained after sequencing are in FASTQ format. The raw paired-end sequences were decontaminated using the Trimmomatic software. The decontamination parameters are as follows: detect and truncate the ambiguous base N. Meanwhile, a sliding window method was used to check the average base quality. When the quality was lower than 20, the high-quality sequence before was intercepted. The decontaminated paired-end sequences were spliced using the FLASH software. The splicing parameters were: the minimum overlap was 10 bp, the maximum overlap was 200 bp, and the maximum mismatch rate was 20%. To ensure the accuracy of the results, accurate decontamination was performed to remove sequences containing ambiguous bases, single-base high-repeat regions (homologous), and sequences with too short lengths. The parameters of accurate decontamination are: remove sequences containing N bases and retain sequences with a base quality fraction Q20 reaching at least 75%. At the same time, the chimera sequence in the sequence was detected and removed using UCHIME. After the sequencing data was preprocessed to generate high-quality sequences, the Vsearch software was used to classify the sequences into multiple OTUs according to the similarity of the sequences. The parameter was that sequences with a sequence similarity of at least 97%. are classified into one OTU unit.

### 2.8 Bioinformatics analysis

Alpha diversity analysis reflects the degree of species diversity in the biological environment. By calculating different alpha diversity indices, the species richness and distribution evenness of the sample are evaluated. In this study, the Shannon and Simpson indices were used to characterize diversity, the Pielou’s evenness index was used to characterize evenness, and the Good’s coverage index was used to characterize coverage.

Beta diversity is the degree of diversity between habitats. It compares the differences of samples in different groups. These difference comparisons often have to be based on the similarity of OTU sequences or the structure of the community (that is, species abundance and distribution), and at the same time consider the evolutionary relationship of OTU sequences and the community structure. Using the ASV/OTU table after rarefaction, and calling the “qiime diversity core-metrics-phylogenetic” command according to the presence or absence of the tree file, the Bray-Curtis distance matrix was calculated, and the PCoA analysis was performed on these distance matrices.

The stacked column chart or simply column chart for species composition was the most commonly used method to represent the species composition of multiple samples. By counting the feature table after singletons are removed, the visualization of the composition and distribution of each sample at seven taxonomic levels including phylum, class, order, family, genus, and species was achieved, and the analysis results are presented in a column chart.

### 2.9 Correlation analysis

The correlation analysis between CA125 and CA199 and the gut microbiota (Top 10) was performed using the genecloud tools, a free online platform for data analysis (https://www.genescloud.cn).

### 2.10 Phylogenetic investigation of communities by reconstruction of unobserved states (PICRUSt2) functional potential prediction

The 16S rRNA gene sequence was predicted with PICRUSt2 in the KEGG databases (https://github.com/picrust/picrust2/). Briefly, first align the 16S rRNA gene sequence to construct an evolutionary tree. Then, align the 16S rRNA sequence with the reference sequence to construct a new evolutionary tree. Use the Castor hidden state prediction algorithm to infer the nearest sequence species of the characteristic sequence, and then obtain the copy number of its gene family; calculate the copy number of the gene family of each sample; finally, map the gene family to various databases to obtain the abundance data of the metabolic pathway in each sample (Langille et al., 2013).

### 2.11 Statistical analysis

SPSS 25.0, GraphPad Prism 8.0 and Excel 2021 were used for statistical calculations and plotting. The significance of the difference between the groups was determined by Student’s *t*-test or Mann-Whitney U, and * *P* < 0.05 was considered significant difference, ** *P* < 0.01 was considered an extremely significant difference, *** *P* < 0.001 was considered an extremely significant difference, ns *P* > 0.05 was considered no significant difference.

## 3 Results

### 3.1 General clinical data of the research objects.

The age of the EMT group was 32.23 ± 5.25, and the age of the CTL group was 31.40 ± 3.75, showing no significant difference between the groups (*P* = 0.564); the BMI of the EMT group was 22.53 ± 3.15 kg/m^2^, and the BMI of the CTL group was 21.14 ± 1.94 kg/m^2^, showing no difference between the groups (*P* = 0.096). The average number of pregnancies in the EMT group was 1.32 ± 0.15, and the average number of pregnancies in the CTL group was 1.30 ± 0.11, with no difference between the groups (*P* = 0.101). The average number of deliveries in the EMT group was 1.05 ± 0.18, and the average number of deliveries in the CTL group was 0.98 ± 0.15, with no difference between the groups (*P* = 0.213) (**Table 1**).

**Table 1:** Clinical data in the EMT and CTL groups.

Group	n	age	BMI (kg/m^2^)	number of pregnancies	number of deliveries
EMT group	18	32.23 ± 5.25	22.53 ± 3.15	1.32 ± 0.15	1.05 ± 0.18
CTL group	18	31.40 ± 3.75	21.14 ± 1.94	1.30 ± 0.11	0.98 ± 0.15
*P*-value	–	0.564	0.096	0.101	0.213

### 3.2 The expression levels of serum markers CA125 and CA199.

Through statistical analysis: (1) The average CA125 of patients in the EMT group was 61.37 ± 13.56 U/mL, and the average CA125 of the healthy group was 17.11 ± 2.87 U/mL; the difference was statistically significant (*P* < 0.001). (2) The average CA199 of patients in the EMT group was 61.55 ± 14.44 U/mL, and the average CA199 of the healthy group was 13.83 ± 2.86 U/mL; the difference was statistically significant (*P* < 0.001) (**Table 2**).

**Table 2:** The expression levels of serum CA125 and CA199 in the EMT and CTL groups.

Group	n	CA125 (U/ml)	CA199 (U/ml)
EMT group	18	61.37 ± 13.56	61.55 ± 14.44
CTL group	18	17.11 ± 2.87	13.83 ± 2.86
*P*-value		0.000	0.000

### 3.3 Alpha and beta diversity of intestinal microbiota

**Figure 1:**
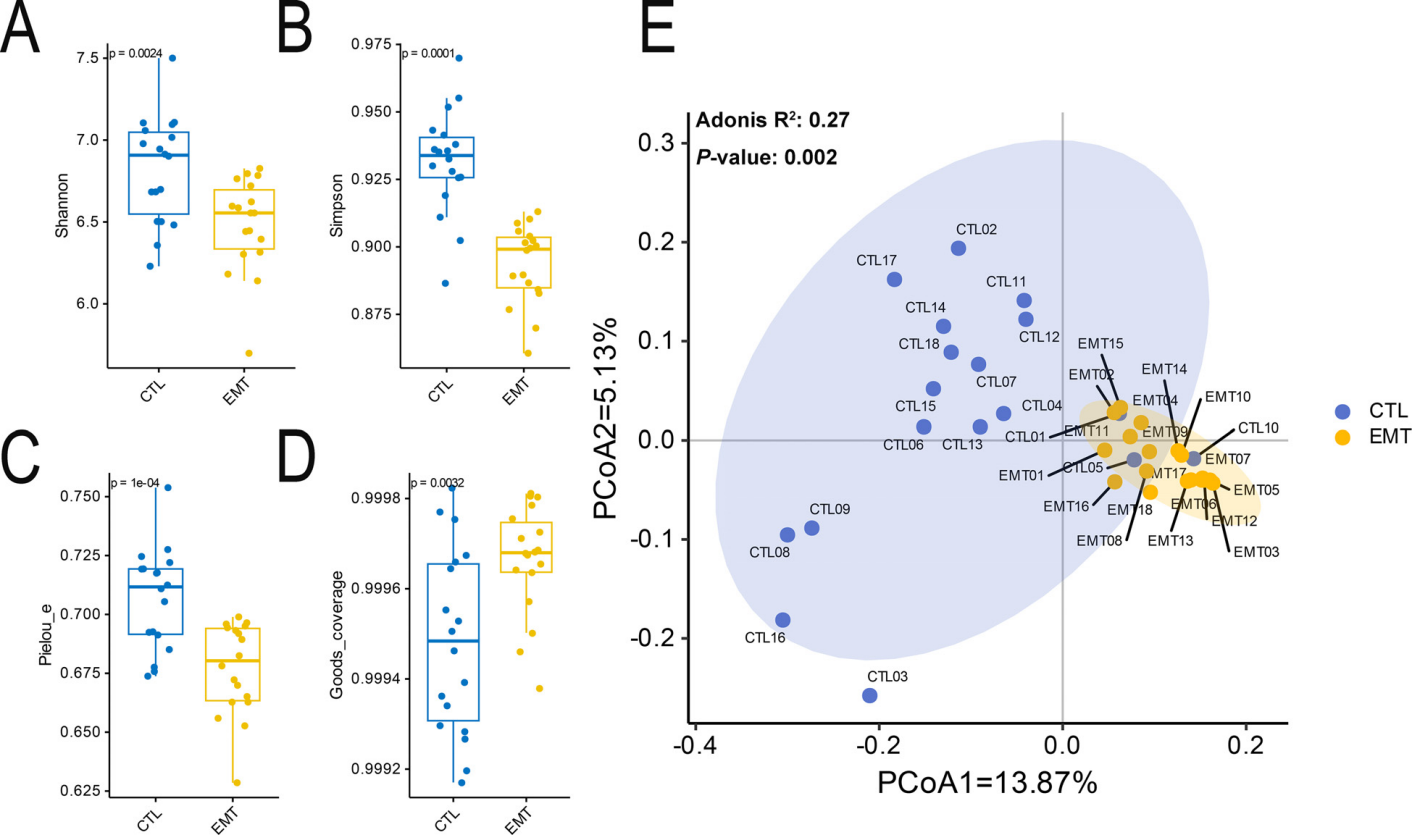
Alpha and Beta diversity of intestinal microbiota in EMT and CTL groups. **(A)** Shannon’s diversity index. **(B)** Simpson’s diversity index. **(C)** Pielou_e’s diversity index. **(D)** Good’s coverage’s diversity index. **(E)** Principal coordinates analysis (PCoA).

The alpha diversity results showed that the diversity and evenness of the intestinal microbiota in the EMT group were significantly lower than those in the CTL group (*P* < 0.05) (**Figures 1A-C**). Additionally, the coverage of the intestinal microbiota in the EMT group was significantly higher than that in the CTL group (**Figure 1D**) (*P* < 0.05). The Beta diversity results showed that samples in the EMT group were relatively close in distance, indicating relatively small differences in community composition among them. In contrast, samples in the CTL group were relatively far apart, suggesting relatively large differences in community composition. (**Figure 1E**).

### 3.4 Species composition of intestinal microbiota

**Figure 2:**
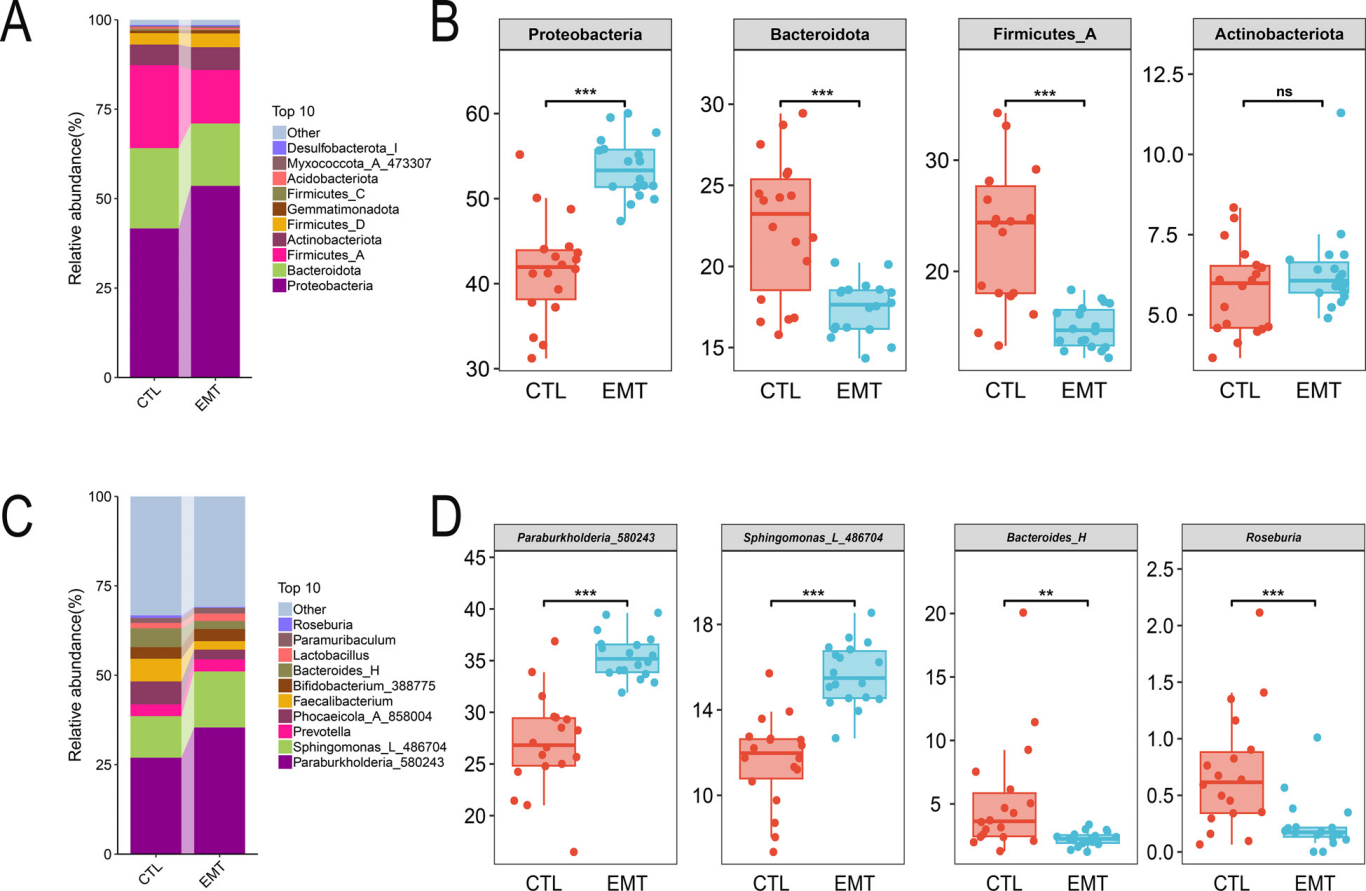
Species composition of intestinal microbiota in EMT and CTL groups. **(A, B)** Species composition of gut microbiota at the phylum level. **(C, D)** Species composition of gut microbiota at the genus level. ** represents *P* < 0.01, indicating a very significant difference. *** represents P < 0.001, indicating an extremely significant difference. ns represents P >0.05, indicating no significant difference.

At the phylum level, Proteobacteria, Bacteroidota, Firmicutes_A and Actinobacteriota were the dominant in both the EMT group and the CTL group (**Figure 2A**). Among them, the relative abundances of Proteobacteria in the EMT group were significantly higher than those in the CTL group (*P* < 0.001). Meanwhile, the relative abundances of Bacteroidota and Firmicutes_A in the EMT group were significantly lower than those in the CTL group (**Figures 2B**) (*P* < 0.001). At the genus level, *Burkholderiales_592524*, *Bacteroidales* and *Roseburia* were the dominant genera in the EMT group and the CTL group (**Figure 2C**). Among them, the relative abundances of *Burkholderiales_592524* and *Sphingomonadales* in the EMT group were significantly higher than those in the CTL group (*P* < 0.001). In contrast, the relative abundances of *Bacteroides_H* and *Roseburia* in the EMT group were significantly lower than those in the CTL group (**Figures 2D**) (*P* < 0.01 or *P* < 0.001).

### 3.5 Species differences and biomarker species

**Figure 3:**
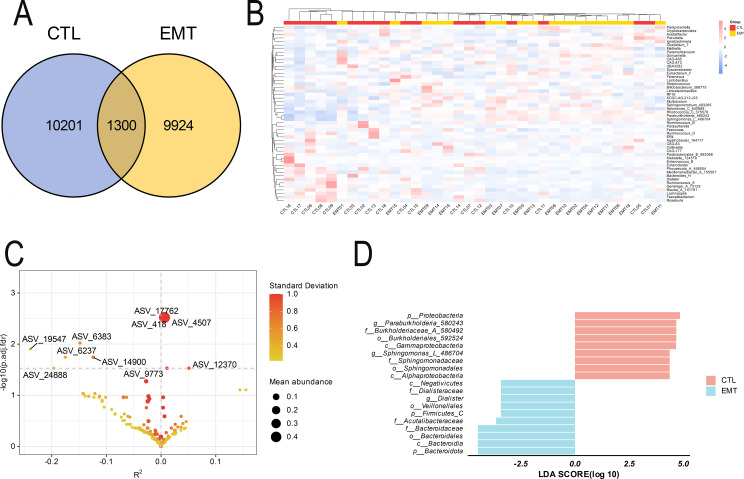
Species differences and marker species of intestinal microbiota in EMT and CTL groups. **(A)** Venn Diagram. **(B)** Species composition heatmap. **(C)** Zicoseq analysis (Standard Deviation is the standard deviation of OTU/ASV abundance, and a larger value indicates a more discrete abundance of OTU/ASV. Mean abundance is the average abundance of OTU/ASV. The Y-axis is - log10 (p.adj. fdr), and a larger value indicates a more significant difference. The X-axis is R2, a negative value indicates that the abundance of the CTL group is greater than that of the EMT group, and a positive value indicates that the abundance of the CTL group is smaller than that of the EMT group (R^2^ (percentage of variance explained) is an effect size measure and can be used to assess the association strength between the taxa abundance and the covariate of interest while adjusting for other covariates). **(D)** LEfSe analysis (The bar chart mainly displays the LDA score distribution of species with significant differences (|LDA score| > 2.0), that is, the Biomaker with statistical differences. The color of the bar chart represents their respective groups, the length represents the LDA score, and the longer the length, the more significant the difference in the species).

The Venn diagram results showed that the EMT group and the CTL group exclusively had 9924 and 10201 ASV sequences respectively, and they shared 1300 ASV sequences (**Figure 3A**). The results of the species composition heatmap (based on the genus level) showed that in the EMT group, the relative abundances of *Muribaculum*, *Sphingomicrobium_483265*, *Halomonas_C_640989*, *Rhodococcus_C_375578*, *Paraburkholderia_580243*, *Sphingomonas_L_486704* were higher than those in the CTL group. Meanwhile, the relative abundances of *Bacteroides_H*, *Dialister*, *Ruminococcus_E*, *Gemmiger_A_73129*, *Blautia_A_141781*, *Lachnospira*, *Faecalibacterium* and *Roseburia* in the EMT group were lower than those in the CTL group (**Figure 3B**). The analysis results of ZicoSeq revealed that the abundances of ASV_17762 (*g_Sphingomonas_L_486704*), ASV_418 (*g_Paraburkholderia_580243*) and ASV_4507 (*g_Sphingomonas_L_486704*) in the EMT group were significantly higher than those in the CTL group. Meanwhile, the abundances of ASV_6383 (*g_Fusicatenibacter*) and ASV_14900 (*g_Roseburia*) in the EMT group were significantly lower than those in the CTL group (**Figure 3C**, **Supplementary Table S1**). The LEfSe analysis results further revealed that p_Proteobacteria, *g_Paraburkholderia_580243*, f_Burkholderiaceae_A_580492, o_Burkholderiales_592524, c_Gammaproteobacteria, *g_Sphingomonas_L_486704*, f_Sphingomonadaceae, o_Sphingomonadales and c_Alphaproteobacteria were the significantly different species in the CTL group. On the other hand, p_Bacteroidota, c_Bacteroidia, o_Bacteroidales, f_Bacteroidaceae, f_Acutalibacteraceae, p_Firmicutes_C, o_Veillonellales, *g_Dialister*, f_Dialisteraceae and c_Negativicutes were the significantly different species in the EMT group (**Figure 3D**).

### 3.6 The correlation between gut microbiota and CA125, CA199

**Figure 4:**
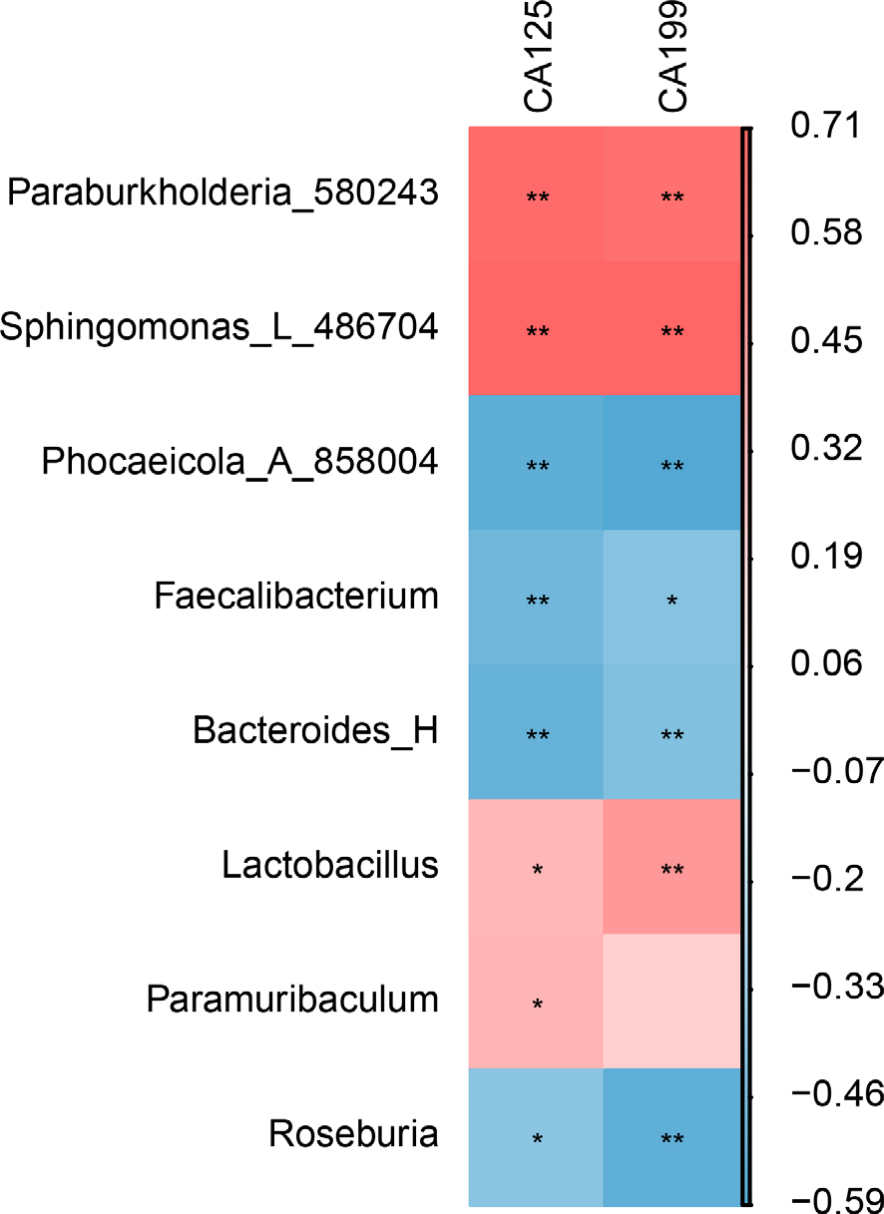
Correlation analysis between gut microbiota and EMT markers (CA125 and CA199). * represents *P* < 0.05, indicating a significant difference. ** represents *P* < 0.01, indicating an extremely significant difference.

The correlation analysis results show that CA125 and CA199 are significantly positively correlated with *Burkholderiales* and *Sphingomonadales*. In contrast, they are significantly negatively correlated with *Bacteroidales*, *Oscillospirales*, and *Roseburia* (**Figure 4**).

### 3.7 PICRUSt2 functional potential prediction

**Figure 5:**
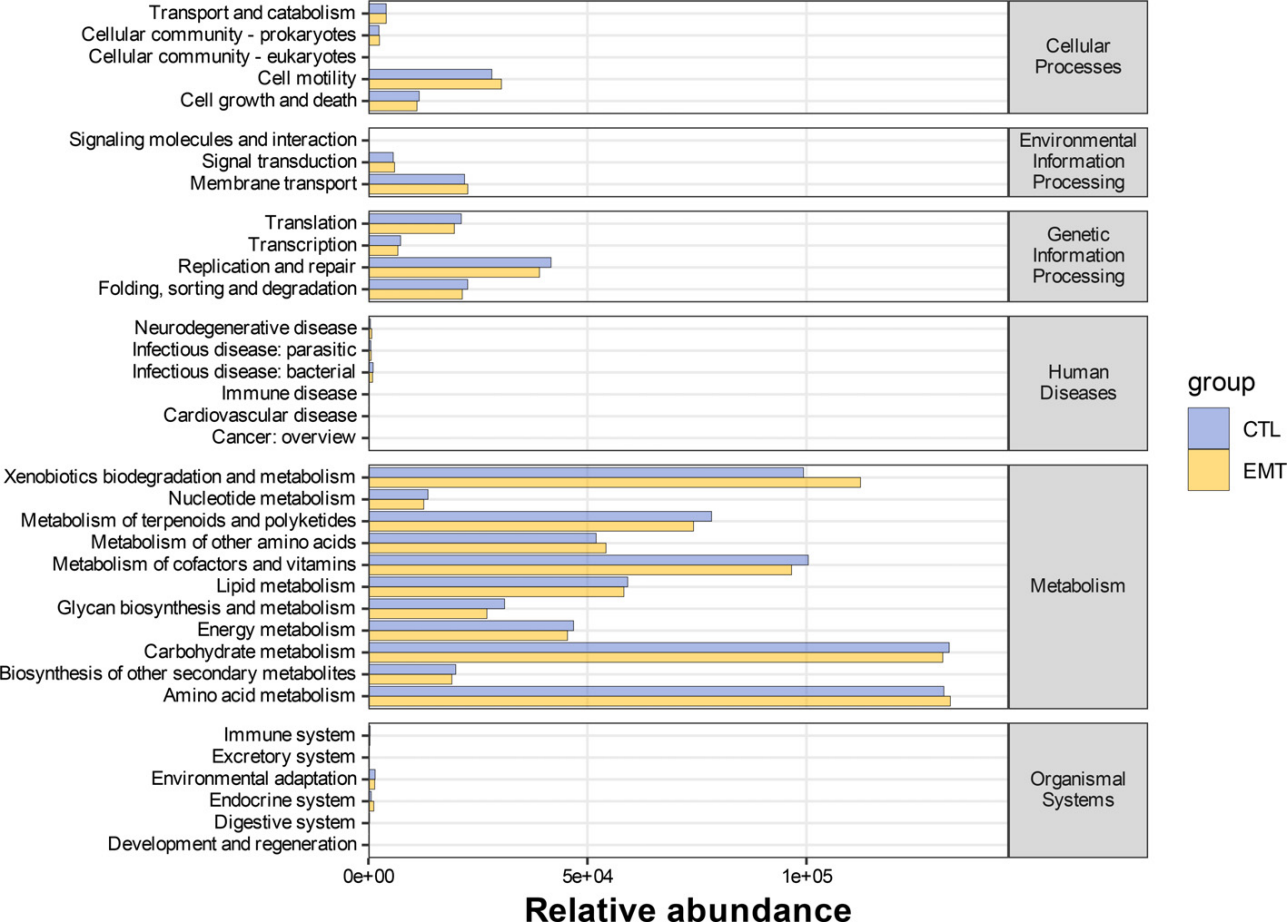
Statistical results of KEGG metabolic pathways involved in gut microbiota (The horizontal axis represents the abundance of functional pathways (in units of millions of KO), the vertical axis represents the functional pathways in the second classification level of KEGG, and the rightmost axis represents the first level pathway to which the pathway belongs).

The KEGG metabolic pathway results indicated that both the EMT group and the CTL group were mainly involved in pathways such as metabolism, genetic information processing, environmental information processing and cellular processes. Specifically, the relative abundance in the cell motility and xenobiotics biodegradation and metabolism pathways in the EMT group was higher than that in the CTL group, while the relative abundance in the translation, replication and repair, folding, sorting and degradation, metabolism of terpenoids and polyketides and metabolism of cofactors and vitamins pathways in the EMT group was lower than that in the CTL group (**Figure 5**).

## 4 Discussion

In recent years, with the explosive expansion of knowledge about the human microbiome, a growing number of human and animal experiments have confirmed that there are significant differences in the microbiota of the intestines, reproductive tracts, and peritoneum. These differences exist between EMT patients and animal models and healthy control groups (Leonardi et al., 2020; Salliss et al., 2021). Although the causal relationship between these changes in microbiota and EMT remains unclear, current research evidence indicated the existence of a two-way relationship between the two (Chadchan et al., 2019). Some researchers have put forward a new hypothesis that it may be that the changes in certain microbiota have damaged the tolerance of the entire immune system, thereby leading to a subclinical inflammatory state and inducing the development of EMT (Tai et al., 2018; Thomas et al., 2018). Given the relatively limited number of reports on the intestinal microecology of EMT patients, this study used fecal samples from EMT patients and healthy people to analyze and compare the intestinal microbial community and diversity, and analyzed its correlation with EMT markers (CA125 and CA199).

As the largest microbiome in the human body, the intestinal microbiome is predominantly composed of two dominant phyla, Bacteroidetes and Firmicutes, which are predominant bacteria in healthy populations. Meanwhile, Proteobacteria is widely regarded as a harmful microbiota in the intestinal microbiome (Guan et al., 2018). A systematic review of the microbial characteristics of EMT found that at the phylum level, Actinobacteria, Firmicutes, Proteobacteria, and Verrucomicrobia were significantly increased in the intestines, while Bacteroidetes was significantly reduced, indicating that EMT is related to the increased number of different microbiota such as Proteobacteria, Enterobacteriaceae, Streptococcus, and Escherichia coli (Leonardi et al., 2020). Ata found through the study of the microbiota at three sites of the intestines, cervix, and vagina of patients with stage III/IV EMT that the intestinal microbial diversity of patients was reduced, and the ratio of Shigella/Escherichia coli was increased (Ata et al., 2019). Huang et al. found that the microbiota of the intestines, peritoneal fluid, and cervix of EMT patients were significantly different from those of healthy women. The depletion of protective microorganisms in the feces of EMT patients and the increase of the abundance of pathogenic bacteria in the peritoneal fluid. It was identified that Ruminococcus and Pseudomonas could be used as potential biomarkers in the intestines and peritoneum, respectively, and it was believed that the intestinal microbiota played a more important role in diagnosing EMT than the cervical microbiota (Huang et al., 2021). Ruminococcaceae produce butyrate, a short-chain fatty acid with anti-inflammatory properties (Tomova et al., 2019). A decrease in Ruminococcaceae could lead to reduced butyrate production, potentially exacerbating inflammation in EMT. Ruminococcaceae may also modulate the immune system (Schirmer et al., 2019). Imbalances in their population could affect immune responses and contribute to the pathogenesis of EMT. Food rich in complex carbohydrates and resistant starch may favor the growth of Ruminococcaceae (Dahl et al., 2020). Incorporating these foods into the diet could potentially increase butyrate production and improve gut health, which may have a positive impact on EMT risk management. Other studies have found that Prevotellaceae is a high-risk factor for EMT (Fu et al., 2023). An increase in Prevotellaceae may potentially contribute to the development of EMT. Prevotellaceae could influence the immune system and inflammatory processes in the body. In EMT, chronic inflammation is a key feature. Changes in Prevotellaceae populations might modulate immune responses and contribute to the inflammatory environment that promotes the growth and progression of EMT lesions (Ji et al., 2023).

In an effort to further explore the causal relationship between intestinal microbes and EMT, researchers have established an animal model of EMT. Yuan et al. induced the establishment of an EMT mouse model by intraperitoneal injection of endometrial tissue and monitored the composition of intestinal microbes. After 42 d of modeling, it was found that the intestinal bacteria in the EMT mice were significantly changed. Specifically, the abundance of Firmicutes and Actinobacteria increased, while the abundance of Bacteroidetes decreased, indicating that EMT induced intestinal microbial dysregulation (Yuan et al., 2018). Chadchan found that after treatment with broad-spectrum antibiotics or metronidazole, the EMT lesions in mice with depleted intestinal microbes were significantly reduced and the inflammation was relieved. Further, after oral gavage of feces from EMT mice to these mice, it was found that the EMT lesions and inflammation in their bodies were restored. Interestingly, when the feces of healthy mice were orally gavaged to these mice, the restoration of EMT lesions was not observed (Chadchan et al., 2019, 2023). These results clearly demonstrate the interrelationship between EMT and intestinal microbiota. The microbes in the feces of EMT mice can induce EMT and inflammation, highlighting the crucial role of intestinal microbes in the growth of EMT.

EMT is an estrogen-dependent chronic inflammatory disease. The immune-inflammatory response serves as the pathological basis (Puente et al., 2016). The microbiota may participate in the pathological mechanism of EMT through multiple pathways, including mediating inflammatory responses, regulating immune responses, participating in estrogen regulation, and interfering with metabolic activities (Walther-António et al., 2016; Ata et al., 2019; Huang et al., 2021). In this study, CA125 and CA199 have a significant negative correlation with *Roseburia* and *Bacteroides*. For instance, *Bacteroides* is crucial for the metabolism and utilization of dietary fiber. This is very important for maintaining the normal function of the intestinal tract (Clemente et al., 2012). In some intestinal diseases, the composition and quantity of *Bacteroides* may change, and by regulating its state, it may help improve the intestinal health status (De Musis et al., 2020). *Roseburia* can produce short-chain fatty acids such as butyric acid, which can suppress the production and release of inflammatory factors and reduce the level of intestinal inflammation (Tamanai-Shacoori et al., 2017). For example, butyric acid can inhibit the activity of some pro-inflammatory cytokines and reduce the occurrence and spread of inflammation (Ge et al., 2023). It can also regulate immune cells: it can affect immune cells in the intestinal tract, such as regulating the balance of T cells, reducing the activity of pro-inflammatory T cell subsets and enhancing the function of anti-inflammatory T cell subsets, thereby maintaining the homeostasis of intestinal immunity and reducing inflammation (Nie et al., 2021). For example, it may promote the increase of regulatory T cells, which helps to suppress excessive immune responses and thereby relieve inflammation. And the occurrence and progression of EMT are closely related to inflammation, and the activation of the inflammatory pathway participates in the pathogenesis mechanism of EMT, and bacteria and their metabolites are involved. The bacterial contamination hypothesis of EMT suggests that endotoxins from bacteria, such as lipopolysaccharide (LPS) and other microbial components, interact with Toll-like receptor 4 (TLR4), mediating the activation of signal pathways to activate macrophages and cause an immune cascade reaction to promote inflammation (Khan et al., 2018), and play an important role in the adhesion and invasion of ectopic and eutopic endometrium (Ying et al., 2018). Immune factors are an important pathogenesis mechanism of EMT. Immune imbalance leads to changes in the local microenvironment of ectopic lesions, which is the main cause of infertility and pain in patients with EMT. EMT is a disease characterized by dysregulated immune responses. There is a disorder of macrophage polarization in the pelvic cavity. Macrophages can help ectopic endometrium avoid immune surveillance and thereby promote the occurrence and development of EMT.

In addition, the intestinal microbiota plays an important role in the regulation of food digestion, immune activation, and intestinal endocrine signaling pathways. The intestinal microbiota can adhere to the surface of intestinal epithelial cells through the production of specific metabolic compounds, such as short-chain fatty acids that adhere to free fatty acid receptors on the surface of intestinal epithelial cells, and interact with neurons or enter the circulatory system to participate in regulating intestinal wall neurons and their development and renewal (Dicks, 2022). A healthy and balanced intestinal microbiota helps maintain mucosal integrity, prevent the translocation of bacteria and their related substances, and promote a normal immune state. In this study, the abundances of metabolism of terpenoids and polyketides, metabolism of cofactors and vitamins, and lipid metabolism metabolic pathways in which the intestinal microbiota of EMT patients participate; while the abundances of xenobiotics biodegradation and metabolism metabolic pathways were abnormally increased. The metabolic products of the intestinal microbiota, such as short-chain fatty acids, bile acids, trimethylamine oxide, and so on, participate in multiple metabolic channels of the host, which may be one of the pathogenesis mechanisms of EMT that promoted by microbiota imbalance (Li et al., 2022). The intestinal microbiota can participate in the occurrence and development of EMT by regulating fatty acid secondary metabolic products (Nie et al., 2018). Research has proved that β-glucuronidase produced by the imbalance of the intestinal microbiota can increase the number and volume of endometrial ectopic lesions (Wei et al., 2023). At the same time, it can affect the transformation of macrophages from MO to M2 subtypes, directly or indirectly promoting the development of EMT. β-Glucuronidase, as an important metabolic product of bacteria, promotes disease progression through interfering with metabolism and participating in hormone regulation, and has been widely recognized (Chan et al., 2016). To sum up, the metabolic products produced by bacteria through synthesis and decomposition are one of the important ways for future basic research to reveal the pathogenesis mechanism of EMT.

## 5 Conclusion

In this study, the increase in relative abundance of *Paraburkholderia-580243* and *Sphingomonas-L-486704*, as well as the decrease in abundance by *Bacteroidees-H* and *Roseburia* may be important factors in the occurrence and development of EMT. In summary, a close correlation exists between the imbalance of gut microbiota and the onset of EMT. The intestinal microbiota has great significance and broad prospects for the prevention, diagnosis and treatment of EMT.

## Data Availability

The raw sequence files were deposited to the National Center for Biotechnology Information Sequence Read Archive with accession number PRJNA1140858.
